# Impact on postpartum hemorrhage of prophylactic administration of oxytocin 10 IU via Uniject^TM^ by peripheral health care providers at home births: design of a community-based cluster-randomized trial

**DOI:** 10.1186/1471-2393-12-42

**Published:** 2012-06-07

**Authors:** Cynthia K Stanton, Samuel Newton, Luke C Mullany, Patience Cofie, Charlotte Tawiah Agyemang, Edward Adiibokah, Niamh Darcy, Sadaf Khan, Alice Levisay, John Gyapong, Deborah Armbruster, Seth Owusu-Agyei

**Affiliations:** 1 Johns Hopkins Bloomberg School of Public Health, Baltimore, Maryland, USA; 2 Kintampo Health Research Centre, Kintampo, Ghana; 3 PATH, Accra, Ghana; 4 Research Triangle Institute, Research Triangle, North Carolina, USA; 5 PATH, Seattle, Washington, USA; 6 University of Ghana, formerly of Ghana Health Service, Health Research Unit, Accra, Ghana; 7 United States Agency for International Development, formerly of PATH, Washington, DC, USA

**Keywords:** Postpartum hemorrhage, oxytocin, uterotonics, randomized trial

## Abstract

**Background:**

Hemorrhage is the leading direct cause of maternal death globally. While oxytocin is the drug of choice for postpartum hemorrhage prevention, its use has generally been limited to health facilities. This trial assesses the effectiveness, safety, and feasibility of expanding the use of prophylactic intramuscular oxytocin to peripheral health care providers at home births in four predominantly rural districts in central Ghana.

**Methods:**

This study is designed as a community-based cluster-randomized trial in which Community Health Officers are randomized to provide (or not provide) an injection of oxytocin 10 IU via the Uniject^TM^ injection system within one minute of delivery of the baby to women who request their presence at home at the onset of labor. The primary aim is to determine if administration of prophylactic oxytocin via Uniject™ by this cadre will reduce the risk of postpartum hemorrhage by 50 % relative to deliveries which do not receive the prophylactic intervention. Postpartum hemorrhage is examined under three sequential definitions: 1) blood loss ≥500 ml (BL); 2) treatment for bleeding (TX) and/or BL; 3) hospital referral for bleeding and/or TX and/or BL. Secondary outcomes address safety and feasibility of the intervention and include adverse maternal and fetal outcomes and logistical concerns regarding assistance at home births and the storage and handling of oxytocin, respectively.

**Discussion:**

Results from this trial will build evidence for the effectiveness of expanding the delivery of this established prophylactic intervention to peripheral settings. Complementary data on safety and logistical issues related to this intervention will assist policymakers in low-income countries in selecting both the best uterotonic and service delivery strategy for postpartum hemorrhage prevention. Results of this trial are expected in mid-2013. The trial is registered at ClinicalTrials.gov: NCT01108289.

## Background

An estimated 350,000 women die each year in childbirth. Progress in reducing maternal mortality has been varied within and between regions, with the least progress in sub-Saharan Africa [1]. The leading direct cause of maternal death worldwide is hemorrhage [2] and it is generally accepted that the majority of these hemorrhages occur during the postpartum period, predominantly from uterine atony. Active management of the third stage of labor (AMTSL)— defined as intramuscular administration of 10 international units (IU) of oxytocin, controlled cord traction, and fundal massage—substantially reduces the risk of postpartum hemorrhage (PPH). A meta-analysis from four facility-based clinical trials showed a 62 % reduction in the risk of PPH (blood loss ≥500 mL) associated with AMTSL [3]. Given the strength of the evidence, the World Health Organization (WHO), the International Federation of Gynecology and Obstetrics (FIGO), and the International Confederation of Midwives (ICM) all recommend AMTSL provided by a skilled birth attendant for all singleton births [4,5].

Oxytocin is a naturally occurring hormone which promotes smooth muscle contraction. When released postpartum it stimulates both the breastmilk let-down reflex and uterine contraction to slow and stop blood loss. Its synthetic analogue is the recommended uterotonic drug of choice for AMTSL, due to its rapid onset of action, minimal side effects, and low cost [5].

In the absence of a skilled birth attendant, WHO recommends that a uterotonic (i.e., oxytocin or misoprostol) should be given by a health worker trained in its use to prevent PPH. Oxytocin is preferred over misoprostol where its use is feasible [6]. While misoprostol has been shown to be effective in preventing PPH at the community level [7-9], in facility-based studies, its efficacy is lower than that achieved by oxytocin and is associated with side effects, including shivering and pyrexia [10]. As Buekens notes, “both drugs have their place in the prevention and treatment of PPH. However, oxytocin is the drug of choice and every effort should be made to make it widely accessible, including at the community level” [11].

Thus, research is needed to assess the safety and feasibility of expanding access to this drug. Expanding provision of oxytocin to peripheral settings raises three main concerns: (1) providers may not be skilled in giving injections, (2) heat exposure may compromise drug integrity/potency, and (3) administration prior to delivery of the baby (i.e., for induction and/or augmentation) outside a fully qualified facility increases risk to mother and baby.

**Figure 1:**
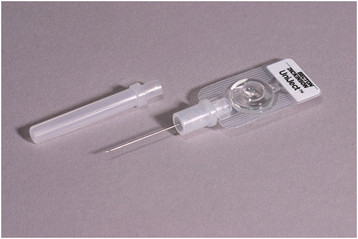
**The oxytocin in Uniject**^**TM**^**injection system.** Source: PATH/Glenn Austin.

The use of oxytocin in the Uniject^TM^ injection system addresses the first of these concerns. The injection system (Figure 1) is an autodisable, “all-in-one” injection system prefilled with the required dosage of drug—in this case, a single dose of oxytocin 10 IU. The injection-ready format reduces wastage, simplifies logistics, and allows use both by lay health personnel who do not normally administer injections and in areas with limited health infrastructure [12]. Oxytocin 10 IU in Uniject^TM^ has been used in observational studies with doctors and midwives at hospitals in Angola and Latin America [13-15], and with birth attendants in the community or lower- level health facilities in Indonesia [16], Mali [17], and Vietnam [18,19]. In these observational studies across multiple settings and cadres of health workers, oxytocin in Uniject^TM^ has been found to be well tolerated and easy to use and has a high level of acceptability among providers.

**Figure 2:**
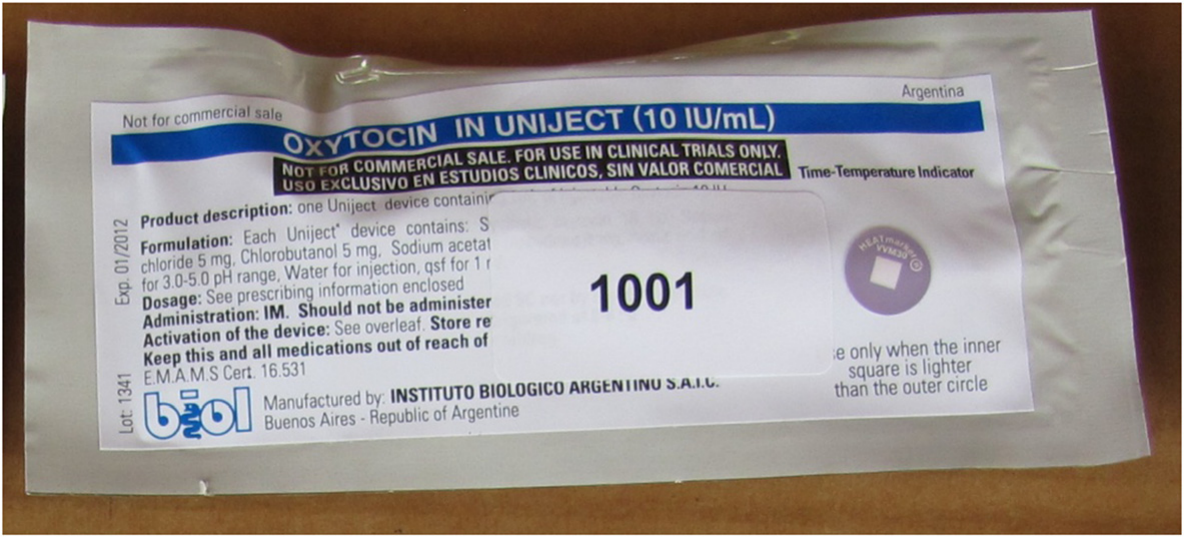
**The Oxytocin in Uniject**^**TM**^**package, including a time-temperature indicator, with an enlarged example of time-temperature indicator colors.** Source: C. Stanton.

The second concern is alleviated by adding a time-temperature indicator (TTI) to the foil pouch containing each Uniject™ injection system (Figure 2). This indicator provides a qualitative measure of cumulative heat exposure by changing color to indicate when the injection system should no longer be used. Studies have shown that oxytocin loses 14 % of its chemically active ingredient when stored at 30°C for one year [20]. The TTI expands the use of oxytocin to the periphery by ensuring that even lay users are able to easily assess if a specific dose is still potent.

Given that a simple injection system is now available which facilitates safe administration of chemically potent oxytocin, a drug known to be effective at PPH prevention when administered by medical personnel in health facilities, the question remains whether this intervention is effective, safe, and feasible in the hands of a minimally trained health care provider assisting home births. This trial, conducted as part of the Oxytocin Initiative project, is designed to address this question.

## Methods/Design

This study is designed as a cluster-randomized community-based trial in which Ghana Health Service (GHS) Community Health Officers (CHOs) are randomized into one of two study arms determining the care that CHOs provide to enrolled women during their home-based deliveries. For the purposes of this trial, all deliveries assisted by an individual CHO constitute a cluster. The trial is designed to determine if intramuscular administration of 10 IU of oxytocin in Uniject™ during the third stage of labor by a CHO will reduce the risk of PPH by 50 % relative to deliveries attended by a CHO who does not provide the prophylactic intervention. This is not, however, a drug efficacy trial. The trial is designed to assess the mode of service delivery.

Implementation of the trial is divided into three phases. The first phase is designed as a month-long Active Preparation Phase in which all field procedures and data collection systems are activated except administration of oxytocin by CHOs. The Comparative Phase marks the initiation of enrollment into the trial and was originally planned to last nine months. The third phase of the trial is a conditional three month Universal Distribution Phase in which all women enrolled in the trial would receive prophylactic oxytocin following delivery of the baby. Implementation of the Universal Distribution Phase of the trial is conditional upon results of the Comparative Phase and feedback from the Data Safety and Monitoring Board.

### Study site

**Figure 3:**
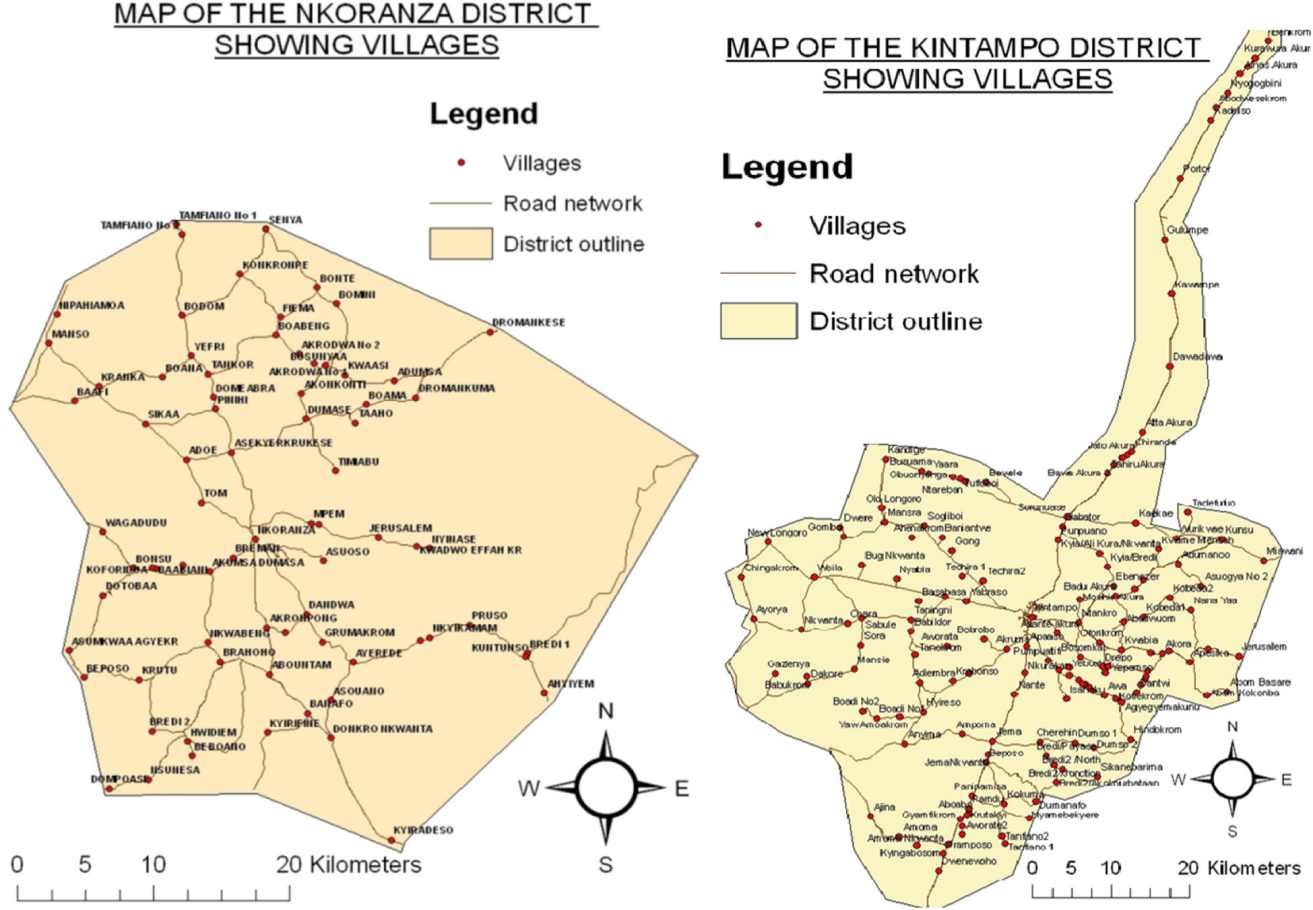
Maps of Nkoranza and Kintampo Districts, Brong-Ahafo Region, Ghana.

The trial is being conducted in four predominantly rural districts of Brong-Ahafo region in central Ghana. The study districts, which are under demographic surveillance by the Kintampo Health Research Centre (KHRC), are Kintampo North and South and Nkoranza North and South (Figure 3), with a combined population of approximately 260,000. KHRC is a field research center operating since 1994 under the GHS and responsible for many large-scale, prospective clinical and community-based trials. The selected districts are an excellent context in which to assess home-based PPH prevention because maternal mortality in this area is high (at approximately 350 to 400 maternal deaths per 100,000 pregnancies [21]) and approximately 30 % of births occur at home.

### Field staff

Field staff for this trial consist of 69 field workers, with at least senior-high school education, dedicated to this study and resident in the communities for which they are responsible, and nine field supervisors. The field workers were provided with bicycles and the supervisors were provided with motorcycles to facilitate movement within their areas of work. All field staff participated in a three-day training prior to the start of the study.

Extensive consultations were required during the design phase of the study between KHRC leadership, study investigators, and GHS staff at both the district and regional levels regarding the involvement of GHS CHOs as the agents to administer the study intervention. CHOs are the centerpiece of the GHS Community-based Health Planning and Services (CHPS) Initiative, which has the goal of expanding access to basic health care for the rural population. CHO services include childhood vaccination, antenatal and postnatal care, family planning outreach, emergency deliveries, and referral for complications during pregnancy and delivery. While CHOs are permitted to administer ergometrine tablets in an emergency for treatment of postpartum hemorrhage, they are not currently permitted to administer injectable uterotonics. Following training, CHOs are posted to a CHPS compound from which their clinical and outreach work is based.

The study facilitated the placement of CHOs into communities in study districts where there were no CHOs and provided resources to improve CHO compounds where these were inadequate. Prior to the start of the study, all CHOs completed a five-day training course and were required to pass a competency-based examination specific to their cluster allocation. Throughout the course of the study, CHOs are provided with some allowance to purchase credit for their mobile phones as well as basic comfort items such as solar lamps and beds. The compounds are also provided with basic hospital equipment such as examination couches and cupboards for storage of medical supplies as a contribution to the expansion of the CHPS initiative. Study-related work by the CHOs is jointly supervised by the GHS and KHRC.

### Randomization

Among the four participating districts, 52 CHO areas were identified and categorized as either “near” (≤ 10 km) and “far” (> 10 km) from a facility with capacity for emergency obstetric care. The categorization resulted in eight strata (4 district x 2 distance), two of which contained an odd number (n = 7, 7) and six of which contained an even number of CHOs (n = 6, 10, 4, 4, 8, 6). A temporary placeholder CHO was added to each of the two strata containing an odd number of CHOs, and all units were randomly allocated to either the oxytocin in Uniject^TM^ group or the Comparison group using the STATA utility “ralloc” [22], stratified on district and distance. This random allocation was repeated 10,000 times. Among the 4,995 allocation sequences that assigned the placeholder CHOs to opposite groups, a single allocation was selected at random, and the two placeholder CHOs were discarded. This resulted in 52 total CHOs evenly allocated overall (26 vs. 26), within each of the six strata with an even number of CHOs, and in a ratio of 4:3 and 3:4 in the two strata with an odd number of CHOs (Table 1).

**Table 1:** Number of CHOs randomly allocated to OIU and comparison groups as a result of randomization procedure stratified on district and distance from facility

**District**	**Near (≤10 km)**		**Far (>10 km)**	
	**Oxytocin in Uniject**^**TM**^	**Comparison**	**Oxytocin in Uniject**^**TM**^	**Comparison**
Kintampo North	3	3	3	4
Kintampo South	5	5	4	3
Nkoranza North	2	2	4	4
Nkoranza South	2	2	3	3

**Figure 4:**
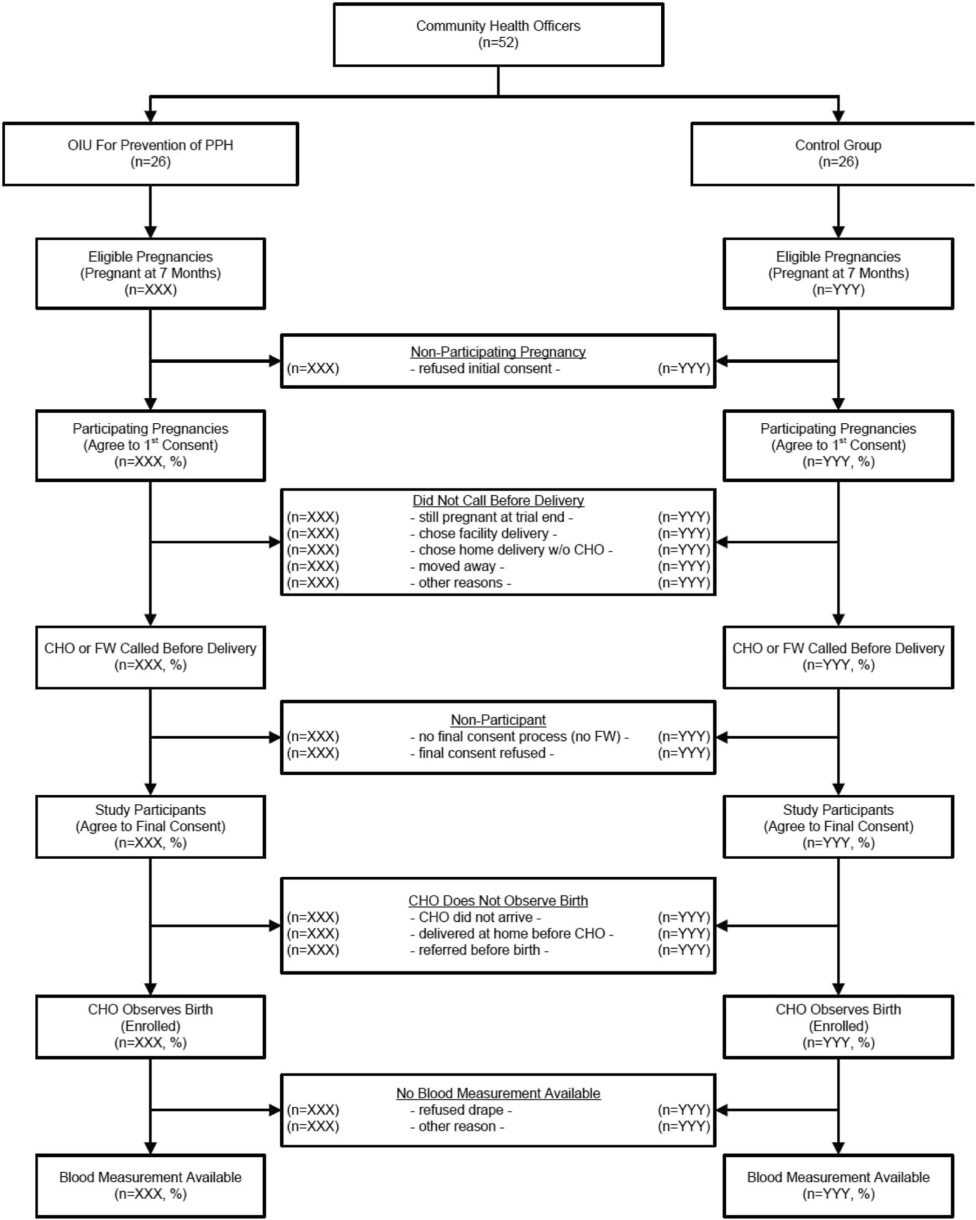
Flowchart of trial design.

Figure 4 describes the flow of all trial activities from randomization through blood loss measurement.

### Procurement of oxytocin in Uniject^TM^

Oxytocin in Uniject™ injection devices with time-temperature indicators were produced by Instituto Biológico Argentino (BIOL), a pharmaceutical producer in Argentina, and imported into Ghana as clinical study supplies after obtaining a clinical sample import permit from the Ghana Food and Drug Board. They are transported to KHRC under cold chain conditions and are distributed from KHRC refrigerators on a weekly or as-needed basis to CHOs, who store them in their compounds in a cool place. Storing oxytocin out of the cold chain is being done to ensure that the intervention is being conducted under conditions as similar as possible to a scaled-up government service. To the best of our knowledge, this study constitutes the first randomized controlled trial in which the oxytocin in Uniject^TM^ injection system has been used.

### Recruitment

Prior to the start of field work, community meetings were held with GHS authorities in various communities, opinion leaders, chiefs, and community members regarding the objectives of the study. KHRC field workers then began identifying on a weekly basis all households with a pregnant woman within their allotted communities. There are no exclusion criteria for this study. As pregnant women at seven months or more gestation are identified, the study is explained to them and they are asked for initial consent to participate in the study. Initial consent indicates that a woman is interested in having a CHO present at her delivery should she be unable to travel to a health facility or should she choose to deliver at home. Field workers remain in contact with women who have initially consented, and where possible, they arrange to introduce the CHO to the pregnant woman’s family. They then wait to see if the woman calls the field worker at the onset of her labor. When field workers are contacted, they phone the CHO and both travel to the woman’s household, although the field worker will generally remain outside of the house. Final consent for full participation is obtained at this time.

### Trial implementation

**Figure 5:**
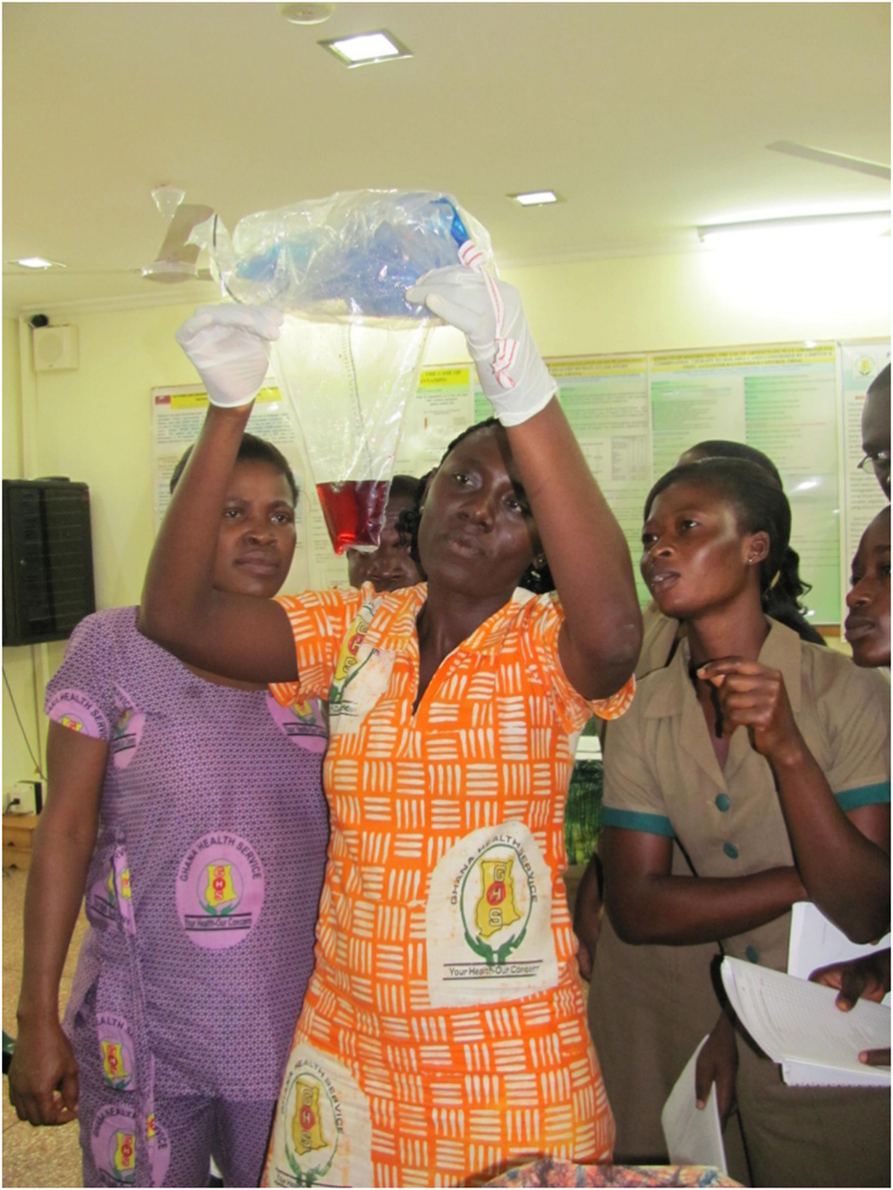
**Training exercise for use of the BRASSS-V blood collection drape.** The individuals pictured gave consent for this image to be published.

Once in the home and after administration of final consent, CHOs in both groups place a BRASSS-V calibrated drape designed to collect postpartum blood under the woman’s body before birth of the baby. Women are asked to remain recumbent, if possible, for one hour if bleeding ceases within one hour, or for two hours if active bleeding continues at one hour. The blood loss measure excludes fluid, urine, and feces passed during the birthing process. All blood collected in the drape is scooped into the pouch. The drape is removed from the woman and held up vertically in order to obtain the blood loss reading (Figure 5). This method has been validated and used in a number of previous and ongoing trials [23]. If a woman in either arm of the trial is actively bleeding when she has lost 400 mL blood, referral is initiated via mobile phone for emergency transport. To identify active bleeding, all CHOs are trained to monitor pulse, uterine tone, and vaginal bleeding every 15 minutes for the first two hours after delivery of the baby. Study-sponsored vehicles and drivers specifically available for this purpose immediately respond to emergency calls. In the event of PPH (defined as ≥ 500 mL blood loss) early treatment with one injection of 10 IU of oxytocin and fundal massage will be provided by the CHO. This same treatment response may also be provided in the event of gushing blood, a uterus that is neither hard nor round, and/or blood clots the size of a lime following delivery of the baby. Palpation for a second twin is done prior to all injections of oxytocin. Emergency referral and transport is available to women and newborns in both arms of the study for any type of complication.

### The intervention

While all CHOs measure blood loss and provide treatment and referral (if necessary), CHOs randomized to the intervention arm provide one injection of 10 IU of oxytocin administered via Uniject^TM^ in the thigh within one minute after delivery of the baby. CHOs in both arms collaborate with the birth attendant to conduct their study-related responsibilities and do not directly manage the delivery.

### Follow-up

Two to three days following delivery, the field worker visits the house of all enrolled women for a follow-up interview with questions regarding the care they received, and in particular, the timing relative to delivery of the baby of any injections they may have received. When necessary, a family member who was present during the birth may assist in responding to these questions. In cases where traditional birth attendants managed the delivery, a similar interview is also conducted with them.

### Sample size estimation

The sample size estimation procedure followed a standard approach for cluster-randomized trials [24]. First, having a priori determined that the desired recruitment period for the comparative phase of the trial would be 9 months, we estimated the expected mean number of observed deliveries per CHO over this time period (mean cluster size = 24), derived from available estimates of (1) the crude birth rate (27 per 1,000), (2) the mean population size covered by a CHO (4,250), and (3) the total proportion of deliveries that would be conducted at home and reachable by CHOs (28 %). Second, in the absence of prior information on the incidence of PPH in this setting, we estimated from a previous community-based trial in rural India that the observed frequency of PPH among women delivering in the presence of comparison CHOs would be approximately 10.0 %, and we assumed a conservatively high variation coefficient (“k” = 0.35) across CHOs. Setting the Type I error rate to 5 % and assuming 10 % loss per cluster, we estimated that 26 CHOs (or 564 pregnancies) would be required within each group (i.e., total = 1128) in order to detect reductions in PPH of 50 % or more with at least 80 % power. After the actual pace of enrollment was established (~August 2011), we re-estimated that the time to reach this initially estimated sample size of 1128 would require 12 months of recruitment.

### Data management

CHOs, KHRC field workers, and supervisors collected data using paper-based forms during their contacts with pregnant women and family members. These included a Background Information form filled at the time of the preliminary consent (approximately seven months gestation); a CHO form filled at and soon after the observed delivery; and follow-up forms with the delivered woman, a family member, and, if present, the traditional birth attendant that assisted at the time of delivery. All forms filled by CHOs and field workers were checked by supervisors for completeness and consistency, and then transferred to the KHRC Computer Center for double data entry into a customized Visual FoxPro database. Consistency, validity, and referential integrity are enforced through automated post-entry checks on the database. Basic enrollment status reports are generated fortnightly.

### Data analysis

Both the interim and final analyses will begin with descriptive information regarding the extent (i.e., total numbers) of enrollment, the dynamic of enrollment over the period of the trial, and variation between the clusters in terms of enrollment. Given the importance of assessing feasibility of this intervention in any scaled-up program, particular focus will be placed on describing the frequency with which women in labor contact the CHO to request her presence and the proportion of these women for whom the CHO arrives before delivery. Reasons for not contacting the CHO or for non-arrival by the CHO will be estimated and compared across the groups. Enrolled women will be defined as those for whom (1) the field worker/CHO arrived at the home prior to delivery, (2) consent to participate was reconfirmed ("final consent"), and (3) the CHO was present at the home of the woman at the time of delivery. Among enrolled women, a range of covariates will be compared to determine if the cluster randomization achieved balance across socio-economic, household, and maternal characteristics.

#### Primary outcome

The primary outcome of this trial is PPH. This outcome was selected based on (1) extended discussions with an international technical advisory group established for the study, which concluded that for policy impact, it would be insufficient to assume a health benefit from this intervention given the use of minimally trained health care providers and use of a new technology; (2) a general consensus that blood loss equal to or greater than 500 mL constitutes PPH; and (3) the availability of a validated tool for the measurement of blood loss in a home setting.

PPH will be defined using three different definitions (Table 2). The strictest definition will be cumulative blood loss of ≥ 500 mL as measured by the BRASSS-V drape at the end of the first hour after birth, or in the case of active bleeding at one hour, at the end of the second hour after birth. Under this definition, all women who have blood loss measured will be included; those with ≥ 500 mL will be included in the numerator and all others with a quantitative measure will be in the denominator. Two further definitions will be examined, given that this strict definition is problematic in two ways. First, some women (in both groups) will be provided a treatment dose either before one hour is complete, or prior to reaching 500 mL; this dose could obscure the total amount of blood loss that might have occurred in the absence of this rapid treatment response. Therefore, a second broader definition will include in the numerator all women with ≥ 500 mL measured blood loss OR women receiving a treatment dose of oxytocin (regardless of final measured blood loss) and will be assessed among all enrolled women. Furthermore, some women may be referred by the CHO for postpartum blood loss, yet not be included in the numerator (or the denominator) of either the prior two definitions. Thus, the final definition will include in the numerator all women with one or more of the criteria (≥ 500 mL blood loss, receipt of a treatment dose, referral for blood loss) and will be assessed among all enrolled women.

**Table 2:** Definitions of the primary outcome: postpartum hemorrhage

**Definition**	**Blood loss ≥ 500 mL**	**Treatment for blood loss**	**Referred for blood loss**	**Numerator (anycriteria met)**	**Denominator**
**1**	✓			- observed blood loss ≥500 mL	Has quantitative measure of blood loss
**2**	✓	✓		- observed blood loss ≥500 mL - received treatment dose	Has quantitative measure of blood loss
**3**	✓	✓	✓	- observed blood loss *≥*500 mL - received treatment dose - CHO referred for blood loss	All enrolled women

Analysis of each of these three definitions will follow an intent-to-treat approach; that is, enrolled women will be included in the analysis according to their cluster allocation, regardless of their specific receipt (or non-receipt) of the assigned intervention. Separately for each definition, the proportion of women in each group meeting the criteria will be estimated, and the risk of the outcome in the intervention group relative to the control group will be calculated (i.e., risk ratio) using a binomial regression model with log link function. Standard errors will be adjusted for the clustered allocation using the generalized estimating equation approach [25], and 95 % confidence intervals will be estimated. If necessary, regression models will be adjusted for any imbalance(s) between the groups, previously identified during the descriptive analysis phase.

#### Secondary outcomes

Given concerns regarding the safety of the intervention and its programmatic feasibility, several secondary outcomes were selected. The predominant safety concern is administration of oxytocin for induction, or more likely, for augmentation of labor. Use of any intramuscular uterotonic drug before the birth of the infant is regarded as dangerous because it is not possible to adjust the dosage if it causes hyperstimulation and cannot be adapted to the level of uterine activity, as is possible with the monitored administration of intravenous oxytocin in health facilities [26]. Hyperstimulation of the uterus can lead to uterine rupture, fetal asphyxia or fetal demise [27,28]. Thus, the key secondary outcome for this trial is mistimed use of oxytocin, and will be defined as any use of oxytocin prior to delivery of the (last) baby. The proportion of deliveries in which a CHO, traditional birth attendant, or pregnant woman/family member reports that oxytocin in Uniject^TM^ was given prior to delivery of the (last) baby will be estimated, and a 95 % confidence interval constructed. If appropriate, this outcome will be examined separately by allocation group and compared using standard methods (again, adjusting standard errors for cluster randomization).

Other safety outcomes include individual and composite estimates of the frequency of adverse maternal, fetal, and neonatal events, including maternal deaths, stillbirths, early neonatal deaths, birth asphyxia, need for newborn resuscitation, uterine rupture, and referral and/or transport to a higher-level facility. Among participating women, comparisons will be made for all deliveries across both arms of the trial and also restricted to deliveries for which oxytocin was given before delivery of the baby. For each comparison (and the composite measure), proportions will be estimated, and 95 % confidence intervals constructed. Comparisons across groups will utilize binomial regression with a log link function, will account for clustered allocation using GEE, and will adjust for imbalance as necessary. Analyses of feasibility outcomes will be descriptive in nature (estimating proportions, means, etc.). These include, among others, the proportion of deliveries in which the CHO arrives late, the mean time between call and arrival of the CHO, proportion of participating pregnancies (initial consent given) that become participants (final consent given), and the proportion of oxytocin in Uniject^TM^ injection systems disposed of properly.

### Data Safety and Monitoring Board and interim analyses

An independent Data Safety and Monitoring Board (DSMB) consisting of national and regional experts was established. Stopping guidelines for efficacy were agreed upon by the DSMB members and the investigating team prior to the trial, using Lan-DeMets boundaries and nominal p-values for group sequential analyses estimated using O’Brien-Fleming spending function [29,30]. A single interim analysis was conducted at ~47 % of the initially planned overall recruitment (December 2011). At the interim analysis, the DSMB recommended continuation of the trial, and suggested extending total enrollment to the extent possible given available resources. Based on this recommendation, the trial was approved for a sample size of 1850 women with data collection continuing for a total of 18 months of recruitment.

### Approvals and registration

The study protocol was approved by the KHRC and GHS Ethical Review Committees, the Institutional Review Board of the Johns Hopkins Bloomberg School of Public Health (#00002673), and the Research Ethics Committee of PATH (#547). The protocol is reviewed and reapproved on an annual basis. The trial is registered at ClinicalTrials.gov: NCT01108289.

## Discussion

Programmatic responses to PPH prevention must take into account contextual issues, including health care provider skills, location, and authorizations for use of specific drugs and modes of administration. Drug effectiveness, PPH treatment options, drug and service delivery costs, and logistical constraints are all important considerations. This trial is designed to address many of these questions for prophylactic oxytocin administered via the Uniject^TM^ injection system.

PPH (defined via blood loss measurement or clinical signs) was selected as the primary outcome of the study for reasons described above. Blood loss data and clinical signs are being used as triggers to initiate early treatment of PPH, and in the case of clinical signs, treatment is initiated without regard to blood loss. Given that PPH is extremely time-sensitive, CHOs are not skilled birth attendants, infrastructure in many areas is poor, and some study households are in remote locations, waiting to initiate treatment until blood loss reached 500 mL or more (as has been done in some health facility-based trials [31,32] was not considered ethically acceptable. Consequently, the low threshold required for early treatment of PPH for some women may have influenced observed blood loss. This is the basis for including broader definitions of blood loss described above and included in Table 2.

The priority for this study design is to conduct a methodologically rigorous study that ensures the safety of all study participants and that yields valid results regarding the risks and benefits of expanding use of a known, effective lifesaving drug for prevention of PPH. Results of this trial are expected in 2013, and will inform national and international PPH prevention policy and guideline development.

## Competing interests

The authors declare that they have no competing interests.

## Authors’ contributions

Important contributions to trial design were made by CS, LCM, SN, OA, JG and DA. The first draft of the manuscript was jointly developed by CS, LCM, SN, SK, AL, and ND. PC and SK are co-investigators and all authors provided editing, assisted in the finalization of the draft, and approved the final manuscript.

## Pre-publication history

The pre-publication history for this paper can be accessed here:

http://www.biomedcentral.com/1471-2393/12/42/prepub
